# Transcriptomics, proteomics, and metabolomics interventions prompt crop improvement against metal(loid) toxicity

**DOI:** 10.1007/s00299-024-03153-7

**Published:** 2024-02-27

**Authors:** Ali Raza, Hajar Salehi, Shanza Bashir, Javaria Tabassum, Monica Jamla, Sidra Charagh, Rutwik Barmukh, Rakeeb Ahmad Mir, Basharat Ahmad Bhat, Muhammad Arshad Javed, Dong-Xing Guan, Reyazul Rouf Mir, Kadambot H. M. Siddique, Rajeev K. Varshney

**Affiliations:** 1 Guangdong Key Laboratory of Plant Epigenetics, College of Life Sciences and Oceanography, Shenzhen University, Shenzhen, 518060 China; 2 Department for Sustainable Food Process, Università Cattolica del Sacro Cuore, Via Emilia Parmense 84, 29122 Piacenza, Italy; 3 Institute of Environmental Sciences and Engineering, School of Civil and Environmental Engineering, National University of Sciences and Technology, Islamabad, Pakistan; 4 Department of Plant Breeding and Genetics, Faculty of Agricultural Sciences, University of the Punjab, Lahore, Pakistan; 5 Department of Biotechnology, Modern College of Arts, Science and Commerce, Savitribai Phule Pune University, Ganeshkhind, Pune, 411016 India; 6 State Key Laboratory of Rice Biology, China National Rice Research Institute, Chinese Academy of Agricultural Sciences (CAAS), Hangzhou, China; 7 WA State Agricultural Biotechnology Centre, Centre for Crop and Food Innovation, Food Futures Institute, Murdoch University, Murdoch, WA 6150 Australia; 8 Department of Biotechnology, School of Life Sciences, Central University of Kashmir, Ganderbal, India; 9 Department of Bio-Resources, Amar Singh College Campus, Cluster University Srinagar, Srinagar, JK India; 10 Zhejiang Provincial Key Laboratory of Agricultural Resources and Environment, Institute of Soil and Water Resources and Environmental Science, College of Environmental and Resource Sciences, Zhejiang University, Hangzhou, China; 11 Division of Genetics and Plant Breeding, Faculty of Agriculture, Sher-e-Kashmir University of Agricultural Sciences and Technology (SKUAST), Srinagar, Kashmir, India; 12 The UWA Institute of Agriculture, The University of Western Australia, Perth, WA Australia

**Keywords:** Artificial intelligence, Bioinformatic tools, Climate change, Defense responses, Environmental pollution, Metal toxicity, Omics approaches

## Abstract

The escalating challenges posed by metal(loid) toxicity in agricultural ecosystems, exacerbated by rapid climate change and anthropogenic pressures, demand urgent attention. Soil contamination is a critical issue because it significantly impacts crop productivity. The widespread threat of metal(loid) toxicity can jeopardize global food security due to contaminated food supplies and pose environmental risks, contributing to soil and water pollution and thus impacting the whole ecosystem. In this context, plants have evolved complex mechanisms to combat metal(loid) stress. Amid the array of innovative approaches, omics, notably transcriptomics, proteomics, and metabolomics, have emerged as transformative tools, shedding light on the genes, proteins, and key metabolites involved in metal(loid) stress responses and tolerance mechanisms. These identified candidates hold promise for developing high-yielding crops with desirable agronomic traits. Computational biology tools like bioinformatics, biological databases, and analytical pipelines support these omics approaches by harnessing diverse information and facilitating the mapping of genotype-to-phenotype relationships under stress conditions. This review explores: (1) the multifaceted strategies that plants use to adapt to metal(loid) toxicity in their environment; (2) the latest findings in metal(loid)-mediated transcriptomics, proteomics, and metabolomics studies across various plant species; (3) the integration of omics data with artificial intelligence and high-throughput phenotyping; (4) the latest bioinformatics databases, tools and pipelines for single and/or multi-omics data integration; (5) the latest insights into stress adaptations and tolerance mechanisms for future outlooks; and (6) the capacity of omics advances for creating sustainable and resilient crop plants that can thrive in metal(loid)-contaminated environments.

## Introduction

Climate change and agricultural production are inextricably linked. Climate change produces various abiotic stresses, including rising global temperatures, drought, cold/freezing, soil salinity, precipitation patterns, wind patterns, waterlogging, metal(loid)s, and other climate events, either directly or indirectly attributed to human actions (Hong et al. 2020; Zandalinas et al. 2021, 2023; Farooq et al. 2022; Benitez-Alfonso et al. 2023). Consequently, understanding the impact of climate change on food safety necessitates a nuanced consideration of the complex interactions that eventually affect the food chain (Shahzad et al. 2021; Zandalinas et al. 2021, 2023; Farooq et al. 2022; Benitez-Alfonso et al. 2023). Among abiotic stresses, metal(loid) toxicity, encompassing elements such as cadmium (Cd), lead (Pb), arsenic (As), copper (Cu), mercury (Hg), nickel (Ni), zinc (Zn), selenium (Se), and iron (Fe), significantly impairs the growth and productivity of food crops (Edelstein and Ben-Hur 2018; Angulo-Bejarano et al. 2021; Raza et al. 2021, 2022; Hassan et al. 2022; Ghuge et al. 2023; Kapoor et al. 2023). Notably, Zn, Se, Fe, and boron (B, an essential micronutrient) are considered beneficial in limited quantities for plant growth and development. These metal(loid)s find their way into soil, water bodies, and air primarily through: (1) anthropogenic activities like mining, petrochemical handling, electronics trash, and municipal waste, (2) natural sources like volcanic events, and (3) modern farming practices, such as excessive fertilizer and pesticide use. Consequently, metal(loid)s have become a global food safety concern as they enter the food chain daily, leading to biomagnification and posing risks to human health and environmental balance. Moreover, these metal(loid)s are abundant in the Earth’s outer layer with manifold advantages but can pollute the environment when in excess (Edelstein and Ben-Hur 2018; Rai et al. 2019; Adimalla 2020; Angulo-Bejarano et al. 2021; Raza et al. 2022; Hassan et al. 2022; Ghuge et al. 2023).

Unlike some pollutants that visibly accumulate in the environment, metal(loid)s can accumulate unnoticed to toxic levels, with plants absorbing them in inorganic or organic forms (https://www.fao.org/fishery/docs/CDrom/aquaculture/a0844t/docrep/008/V3640E/V3640E04.htm). The inorganic form is highly toxic for elements like As and Cu, while organic forms pose the greatest threat for elements like Hg, Pb, and tin (Sn). Soil contamination with metal(loid)s is considered a critical plant warning. Any essential or non-essential traceable metal(loid) exceeding defined safety levels can trigger various abnormalities, ultimately resulting in heightened oxidative stress in plants, inhibited photosynthesis, and a consequent slowdown or cessation in growth (Thakur et al. 2022). Hyperaccumulator plants exhibit varying thresholds for these non-toxic trace elements, depending on the type of metal(loid) and environmental conditions, ranging from 3–10 mg g^–1^ for essential elements and 0.1–1 mg g^–1^ for non-essential elements. In contrast, non-hyperaccumulator species commonly display lower thresholds for these elements, indicating their limited capability to accumulate trace elements, both essential and non-essential. The differentiation in accumulation thresholds highlights the unique metal(loid) tolerance approaches between hyperaccumulator and non-hyperaccumulator species (Patra et al. 2020; Manara et al. 2020; Angulo-Bejarano et al. 2021).

Optimal plant growth and development hinge on the availability of specific mineral nutrients. Essential micro- and macro-nutrients are crucial for fundamental processes such as metabolism, enzyme synthesis and activity, chlorophyll (Chl) functioning, photosynthesis, nitrogen use efficiency, DNA and pigment synthesis (Vymazal 2016; Khan et al. 2016; Patel et al. 2020; Kumar et al. 2021). Some nutritional metal(loid)s, including Cu, Zn, Ni, Fe, and Se, are vital in trace amounts (Karthika et al. 2018; Kumar et al. 2021) but become lethal when accumulated in excess amounts in plants (Karthika et al. 2018; Kumar et al. 2021). Conversely, non-essential toxic metal(loid)s like As, Cd, Pb, and Hg are harmful even in small amounts (Pokorska-Niewiada et al. 2018; Paz et al. 2019; Kumar et al. 2021), causing reduction in growth, biomass, and yield, disruptions in water and nutrient balance, chlorosis, inhibition of the electron transport chain, denaturation of essential enzymes and proteins, generation of reactive oxygen species (ROS), lipid peroxidation, restricted movement of essential nutrients, and even plant death (Edelstein and Ben-Hur 2018; Kosakivska et al. 2021; Raza et al. 2022; Hassan et al. 2022; Thakur et al. 2022; Ghuge et al. 2023; Basit et al. 2023). Due to their toxic nature, certain non-essential metal(loid)s tend to replace beneficial elements in critical enzymes and pigments, disrupting their functionality (Erickson et al. 2019; Kosakivska et al. 2021; Thakur et al. 2022; Ghuge et al. 2023). Thus, plants require these metal(loid)s in small quantities to support normal metabolic processes in the face of climate change.

**Fig. 1:**
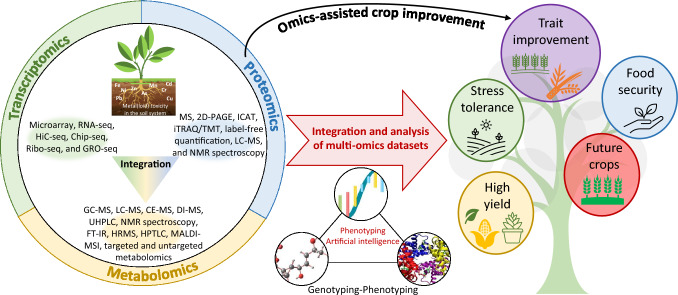
Overview of omics-assisted crop improvement. Integrating three major omics tools, transcriptomics, proteomics, and metabolomics (sometimes combined with high-throughput phenotyping and artificial intelligence), can help toward trait improvement, stress tolerance (single or multiple), development of high-yielding varieties, food security, and development of future crops. Abbreviations: capillary electrophoresis mass spectrometry (CE-MS), chromatin immunoprecipitation-sequencing (Chip-seq), direct-infusion mass spectrometry (DI-MS), Fourier transform ion cyclotron resonance (FT-IR), gas chromatography-mass spectrometry (GC–MS), global run-on sequencing (GRO-seq), high‐throughput chromosome conformation capture-sequencing (HiC-seq), high-resolution mass spectrometry (HRMS), high-performance thin layer chromatography (HPTLC), isobaric tag for relative absolute quantitation/tandem mass tags (iTRAQ/TMT), isotope-coded affinity-tag-based protein profiling (ICAT), liquid chromatography-mass spectrometry (LC–MS), mass spectrometry (MS), matrix-assisted laser desorption/ionization mass spectrometry imaging (MALDI-MSI), nuclear magnetic resonance (NMR) spectroscopy, RNA-sequencing (RNA-seq), ribosome profiling-sequencing (Ribo-seq), two-dimensional polyacrylamide gel electrophoresis (2D-PAGE), ultra-high-performance liquid chromatography (UHPLC)

In recent years, biotechnology-assisted breeding and stress management advances have significantly progressed development of climate-resilient crops for future cultivation. Among modern biotechnological tools, omics techniques such as genomics, transcriptomics, proteomics, metabolomics, ionomics, miRNAomics, and phenomics have emerged as powerful means to unravel the responses and tolerance mechanisms associated with metal(loid) stress in plants (Jamla et al. 2021; Khan et al. 2021; Raza et al. 2022, 2023; Rahman et al. 2022; Sharma et al. 2022; Kumar et al. 2023). Among these, transcriptomics or RNA sequencing, proteomics, and metabolomics hold immense potential in elucidating stress-responsive genes, proteins, metabolites, metabolic pathways, and complex processes during plant development under metal(loid) toxicity, ultimately paving the way for omics-assisted crop improvement (Fig. 1). While numerous studies on stress biology have identified candidate metal(loid)s and their associated genes, proteins, and metabolites across diverse plant species, their application in breeding programs for developing stress-responsive/tolerant plant varieties has yielded few success stories.

While several review articles have explored various omics approaches and provided insights into adaptation and tolerance mechanisms (Singh et al. 2016; Khalid et al. 2019; Jamla et al. 2021; Khan et al. 2021; Yadav et al. 2021; Raza et al. 2022; Rahman et al. 2022), there remains a pressing need for a comprehensive and up-to-date synthesis of the latest research findings in a single, accessible source. Hence, in this review, we comprehensively assess the most recent metal(loid)-mediated omics discoveries across diverse plant species and offer an up-to-date exploration of bioinformatics databases, tools, and pipelines tailored for single and/or multi-omics data. The different sections of this review (1) critically appraise plant responses and adaptation mechanisms to metal(loid) toxicity, (2) curate the latest insights from studies on metal(loid)-mediated transcriptomics, proteomics, and metabolomics studies to lay the foundation for future investigations on adaptation and tolerance mechanisms, (3) highlight the prospects of integrating various omics approaches with machine learning and high-throughput phenotyping (HTP) to improve our understanding of adaptation and tolerance mechanisms, and (4) overview commonly used bioinformatics resources for omics data analysis and integration.

This review serves as a comprehensive repository of knowledge, summarizing the sophisticated ways plants respond and adapt to metal(loid) stress and highlighting the immense potential of omics advances for developing metal(loid)-resilient crop varieties. In short, this review enhances the value of existing literature by offering an integrated and up-to-date perspective on metal(loid) toxicity in plants alongside potential solutions using omics approaches.

## Plant responses and adaptation to metal(loid) toxicity

Plants growing in contaminated areas, including terrestrial and aquatic ecosystems, are continuously exposed to metal(loid)s, resulting in significant yield losses. These substances find their way into the food chain primarily through the uptake and accumulation by crop plants (Edelstein and Ben-Hur 2018; Angulo-Bejarano et al. 2021; Raza et al. 2022; Hassan et al. 2022; Ghuge et al. 2023; Kapoor et al. 2023). Metal(loid)s are translocated within plants through several successive processes, including root uptake, root xylem loading, long-distance translocation via xylem and phloem pathways, and phloem retranslocation (Zhao et al. 2022). During these stages, various membrane transporters, some of which also transport essential or beneficial nutrients like heavy metal-transporting ATPase, zinc-regulated transporter protein (ZIP), and ATP-binding cassette (ABC), are involved in metal(loid) transport (Tao and Lu 2022). However, the physiochemical similarities between metal(loid)s and nutrients can constrain the ability of these transporters to discriminate them (Zhao et al. 2022). For instance, phosphate and arsenate have similar p*K*_a_ values, oxidation states, and ionic charges (Elias et al. 2012).

Metal(loid)s disrupt intracellular homeostasis, triggering oxidation in vital macromolecules like lipids, proteins, genetic material, and enzymes. This oxidative stress results from the overproduction of free radicals and impaired pro-oxidant and antioxidant systems in plant cells (Thakur et al. 2022; Basit et al. 2023). Oxidative-stress-mediated metal(loid) toxicity primarily affects various intracellular organelles and components, including cell membranes, mitochondria, chloroplasts, and some proteins involved in detoxification and metabolism (Kumar et al. 2016; Hasan et al. 2017). Four main mechanisms have been proposed to explain metal(loid) toxicity: (1) competition with other essential micro- and macro-elements, disrupting mineral nutrition (Amari et al. 2017; Vasile et al. 2021); (2) metal(loid) interactions with thiol and carboxyl groups in biomolecules, inhibiting protein and antioxidant enzyme activities (Gulcin and Alwasel 2022); (3) occupation of specific ion-binding sites in proteins, rendering them inactive (Mishra et al. 2017; Li et al. 2022a); and (4) overproduction of ROS, leading to progressive oxidative stress, DNA damage, and ultimately cell death (Georgiadou et al. 2018; Zainab et al. 2021; Ghuge et al. 2023).

**Fig. 2:**
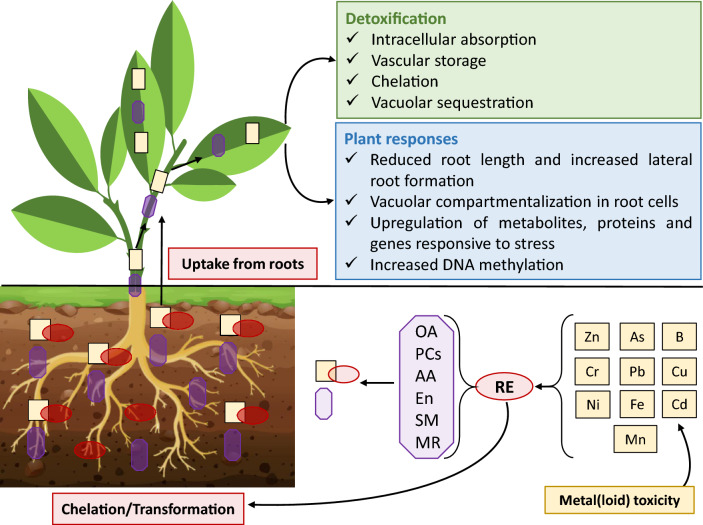
Schematic overview of the mechanisms underlying the restriction, uptake, and detoxification of metal(loid)s in plants and their responses. The uptake of metal(loid)s occurs via root cells, with excessive amounts stimulating root exudation containing various molecules such as OA, PCs, AA, En, SM, and MR. In the rhizosphere, these molecules form complexes with metal(loid)s that restrict their entry into root cells or transform them into less toxic materials. However, metal(loid)s absorbed into root cells can be translocated to the xylem and thus transported to aerial tissues. Abbreviations: arsenic (As), boron (B), chromium (Cr), cadmium (Cd), copper (Cu), enzymes (En), iron (Fe), lead (Pb), manganese (Mn), mycorrhizas (MR), nickel (Ni), organic acids (OA), phytochelatins (PCs), root exudate (RE), secondary metabolites (SM), zinc (Zn)

Like other organisms, plants have evolved strategies to mitigate the harmful effects of metal(loid)s and maintain their homeostasis. These strategies can be broadly categorized into two main mechanisms: (1) restriction mechanism, where plant cells exude specific small molecules into the rhizosphere to chelate metal ions, preventing them from entering cellular components and potentially reducing their toxicity; and (2) detoxification mechanism, where plants absorb high levels of metal(loid) ions, sequestering them in specific internal tissues (Fig. 2) (Gallo-Franco et al. 2020; Ghuge et al. 2023). These mechanisms induce changes at the anatomical, physio-biochemical, and cellular levels. Anatomically, metal(loid)s can influence root development, often reducing root length and increasing lateral root formation as an adaptive response to changes in the root environment (Ronzan et al. 2018; Della Rovere et al. 2022). For example, accumulated Cd induces lateral root proliferation in several crop plants (DalCorso et al. 2008; Piacentini et al. 2020). Likewise, Cu exposure promotes the formation of lateral root primordium in *Arabidopsis thaliana* (Zhao et al. 2021), an important plant strategy to avoid Cu^2+^-induced damage (Lequeux et al. 2010). In some cases, existing root structures undergo cellular changes to prevent excessive metal(loid) accumulation, such as vacuolar compartmentalization in root cells (Sharma et al. 2016) and endomembrane reorganization by compartmentalizing and alterating membrane abilities like permeability, storage, and detoxification (De Caroli et al. 2020).

At the cellular level, modifications occur in cell wall components, including the upregulation of polysaccharides and low-methylesterified pectins, enhancing the cell wall’s ability to bind metal ions (Krzesłowska 2011; Rai et al. 2021). Other metal(loid) detoxification mechanisms involve chelation, metallothioneins (MTs), and vacuolar compartmentalization (Hamim et al. 2018). Organic acids can form metal–ligand complexes (Tahjib-Ul-Arif et al. 2021; Vega et al. 2022) at the root surface or in the cytosol that prevent metal(loid)s from entering vital cellular pathways. Several studies have reported the synthesis and release of low molecular weight organic acids, such as acetic acid and succinate, by roots as an effective strategy for Cd tolerance in the accumulator genotype (Mnasri et al. 2015; Ubeynarayana et al. 2021). For example, low-level As increased the secretion of low molecular weight organic acids like citric, oxalic, and malic acid in the rhizosphere of mangroves (Mei et al. 2021). Likewise, Wang et al. (2022f) showed that acetic acid application improved the remediation performance of oilseed sunflower in Cd-contaminated soils. Phytochelatins (PCs) are synthesized in response to metal(loid) exposure in many crop plants (Loscos et al. 2006; Tennstedt et al. 2009; Fontanini et al. 2018; Zhu et al. 2021). For example, a recent study reported that overexpression of a novel PC synthase gene (*BnPCS1*) improved root growth, decreased peroxidation, and promoted Cd tolerance, accumulation, and translocation in *A. thaliana* (Zhu et al. 2021). PCs also play a role in root-to-shoot translocation of metal ions via the phloem. Defective PC synthesis can change metal ion accumulation patterns and the appearance of sensitive and resistant plants (Zhu et al. 2021). MTs, a superfamily of cys-rich proteins, play an important role in ionomic homeostasis, detoxification, and metal(loid) tolerance (Pan et al. 2018; Li et al. 2022a). Class II MTs, found in plants, can be grouped into four types (MT1–MT4) and have been associated with improved tolerance to metal(loid) toxicity. For instance, functional characterization of an *MT2* gene (*SsMT2*) and its overexpression significantly increased CdCl_2_ tolerance in *Arabidopsis* plants due to increased-Cd accumulation (Jin et al. 2017). Transgenic plants also maintained lower H_2_O_2_ levels than control plants, alleviating Cd toxicity (Jin et al. 2017). In another study, overexpression of the *CarMT1* gene, as a molecular stress marker, enhanced metal(loid) stress adaptive efficiency in chickpea, which improved seed germination and root growth (Kumar et al. 2022).

In addition to these mechanisms, transcriptome-wide analyses have identified genes involved in signal transduction pathways, oxidative-stress-related metabolites (like free radicals), and biosynthetic compounds (e.g., organic acids, polysaccharides, and hormones) as key players in plant responses to metal(loid) stress (Liu et al. 2019). Moreover, plants treated with Cd, Cr, and Pb upregulated genes involved in the oxidative defense system (Alaraidh et al. 2018). In another study, overexpression of rice (*Oryza sativa* L.) class III peroxidase (*OsPRX38*) reduced As accumulation in *A. thaliana* by stimulating apoplastic lignification, increasing antioxidant enzymes (SOD, PRX, and GST), and decreasing H_2_O_2_ and MDA contents (Kidwai et al. 2019). Epigenetic modifications, including DNA methylation and histone acetylation, have also been implicated in metal(loid)-induced responses in plants. For example, *Noccaea caerulescens* (Ni hyperaccumulator) had higher methylation levels than *A. thaliana* (Ni sensitive) when cultivated in Ni-rich soil (Gullì et al. 2018). Another study reported that Cd and Mn influence distinct DNA methylation sites in a concentration-dependent manner (Jing et al. 2022), partly mediated by ROS.

Shafiq et al. (2019) highlighted the importance of DNA methylation and histone acetylation in improving metal(loid) tolerance dynamics mediated by transporters like iron-regulated transporter-like protein and zinc-regulated transporters. Another study reported the intricate relationship between DNA methylation status and adaptive responses in *A. thaliana* exposed to Cd, with upregalted DNA methylation and downregulated expression of DNA demethylase genes *ROS1/DML2/DML3* (RDD) (Fan et al. 2020). Furthermore, inhibiting *RDD*-mediated DNA demethylation enhanced Cd tolerance in *A. thaliana* by increasing iron (Fe) supply through a feedback mechanism (Fan et al. 2020). More recently, Tang et al. (2022) found that Cr-induced DNA methylation coupled with increased antioxidant capacity contributed to shaping plant responses to Cr exposure by influencing gene expression.

## Advances in three major omics approaches for enhancing metal(loid) tolerance

**Fig. 3:**
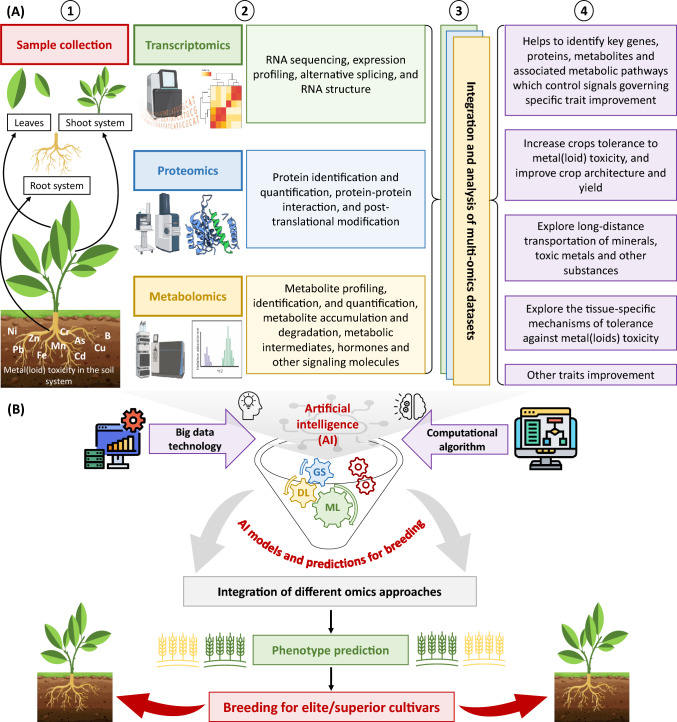
Integrated omics approaches to develop metal(loid)-tolerant crop plants. (**A**) Metal(loid)-toxicity-mediated omics studies comprise four major steps: (1) sample collection against metal(loid) toxicity, (2) design and execution of single or multi-omics tools in one or multiple experiments, (3) integration and analysis of multi-omics datasets, and (4) results interpretation to reveal several key players and mechanisms for developing metal(loid)-tolerant crop plants with improved growth and productivity. (**B**) Inegrating omics data with artificial intelligence to design elite/superior cultivars. Recent innovations in computational algorithm and big data technology have deeply stimulated the growth of artificial intelligence. Using artificial intelligence models, integration of different omics approaches accelerates interpreting how plant phenotypes are accurately predicted and sequentially assists fast-forward breeding for elite/superior cultivars for the future. Abbreviations: Deep learning (DL), genomic selection (GS), machine learning (ML)

Plant responses to metal(loid) toxicity depend on complex and multi-dimensional control system management at the molecular level. Consequently, omics methodologies have become crucial in unraveling the biological interactions and molecular pathways that enhance metal(loid) tolerance. While genomics-assisted breeding has made significant strides (Varshney et al. 2018, 2021), exploring other omics tools (transcriptomics, proteomics, and metabolomics) is important to improve our molecular understanding (Fig. 3). Moreover, single-cell omics approaches have emerged as effective tools for crop breeding to mitigate environmental stresses, including metal(loid) toxicity (Tripathi and Wilkins 2021; Mo and Jiao 2022; Depuydt et al. 2023; Yang et al. 2023; Yu et al. 2023b). These approaches allow for detailed characterizations of individual cells, enabling the detection of subtle changes in gene and protein expression, metabolite accumulation, and metabolic pathways (Giacomello 2021; Tripathi and Wilkins 2021; Mo and Jiao 2022; Lanekoff et al. 2022; Yang et al. 2023; Depuydt et al. 2023; Yu et al. 2023b). Omics approaches can provide valuable insights for identifying key molecular players involved in metal(loid) detoxification and tolerance mechanisms across diverse plant species. Molecular regulators, including genes, RNAs, metabolites, and proteins, and their associated processes, such as replication, transcriptional, translational, post-transcriptional, and post-translational modifications, play pivotal roles in essential plant functions. Furthermore, they are instrumental in shaping plant responses to metal(loid) stress; thus, understanding the regulatory mechanisms at the central dogma level is crucial. In summary, the wealth of scientific knowledge generated by omics tools and databases focused on stress-related signaling pathways, molecular regulators, and coherent mechanisms holds great promise for improving plant survival against metal(loid) toxicity in the ever-changing landscape of climate change.

### Transcriptomic profiling: uncovering molecular mechanisms underlying metal(loid)s tolerance

Transcriptomics has become a critical tool in unraveling the molecular responses of plants to metal(loid) toxicity. This approach allows researchers to study RNA transcripts and gene expression patterns in response to metal(loid) exposure, offering insights into the genes, signaling pathways, and molecular interactions involved (Bhardwaj et al. 2021; Yang et al. 2021; Ullah et al. 2022; Raza et al. 2023; Kumar et al. 2023). Over the years, transcriptomics has evolved in various directions, including single-cell transcriptomics (Almet et al. 2021; Bobrovskikh et al. 2021; Giacomello 2021) and spatial transcriptomics (Larsson et al. 2021; Marx 2021), which offer high-resolution views of cellular mechanisms and interactions (Giacomello 2021; Longo et al. 2021).

Numerous transcriptomics studies have shed light on key genes and metabolic pathways involved in plant responses to metal(loid) stress (Table 1). For instance, a study on Cd-exposed cherry tomato (*Lycopersicon esculentum*) revealed differential expression of genes involved in auxin signaling, antioxidation, and cell wall formation and Cd transporter genes, including *HMA5*, *NRAMP6*, *CAX3*, *ABCC3*, and *PDR1* (Tahjib-Ul-Arif et al. 2021). Rice varieties exposed to Cd exhibited differential gene expression depending on their Cd tolerance. The Cd-tolerant varieties upregulated genes related to oxidation–reduction, catabolism of aminoglycan, iron binding, and heme binding, and downregulated genes related to oxidoreductase activity, photosynthesis, and thylakoid. In contrast, Cd-sensitive varieties upregulated genes involved in heme binding, iron binding, oxidation–reduction, zinc ion transmembrane transport, and tetrapyrrole, and downregulated genes related to carbohydrate metabolism, hydrolyzing of O-glycosyl compounds, and apoplasts (Yu et al. 2021).

**Table 1:** Summary of recent transcriptomics studies performed under metal(loid) toxicity in different plant species

Plant species	Stress condition	Tissue	Key findings	Reference
Soybean (*Glycine max* L.)	Mn (100 µmol L^−1^; 15 d)	Leaves	• Of the 2258 DEGs identified, 1524 were downregulated, and 744 were upregulated • Most of these DEGs were associated with various cellular and binding functions; 49 DEGs regulated hormone signal transduction pathways • AHK, PIF, JAZ, and TGA vital for adaptation to Mn exposure	Xue et al. (2021)
Barley (*Hordeum vulgare* L.)	Co (50 μM) and Cu (50 μM) alone and combined	Roots and shoots	• More DEGs identified for combined exposure to Co and Cu than individual exposures • Of the two barley genotypes tested, the metal-tolerant Yan66 had more DEGs than the metal-sensitive (Ea52)	wa Lwalaba et al. (2021)
Lettuce (*Lactuca sativa* L.)	CuO NPs (100 and 1000 mg L^−1^; 15 d)	Leaves	• Comparative transcriptomic analysis revealed 2270 and 4264 DEGs for 100 and 1000 mg L^−1^ CuO exposure, respectively • Antioxidant enzyme transcript levels, flavonoid content, cell wall structure and components, and hormone alterations were found to be associated with CuO tolerance • Damage to photosynthetic activity and ROS accumulation in leaves	Xiong et al. (2021)
Purple leaf mustard (*Brassica juncea* L.)	Cd (5, 10, 30, and 50 mg kg^−1^; 50 d)	Roots and shoots	• Upregulation of *PME17* (*pectin methylesterases*) and *PME14*, and downregulation of *XTH18* (xyloglucan endotransglucosylase/hydrolase enzymes), *XTH22*, and *XTH23* might have compromised cell wall integrity	Zhang et al. (2021)
Sweet potato (*Ipomoea batatas* L.)	Cd (1 and 5 mg kg^−1^; 4 weeks)	Roots and shoots	• Comparative transcriptomic analysis of two sweet potato cultivars (N88 and X16) revealed that X16 had more DEGs, with 3173 downregulated and 2649 upregulated • DEGs controlled Cd detoxification, cell wall biosynthesis, hormone signal transduction, and glutathione metabolism, with prominent DEGs identified as *AuX*_*1*_*, CAT, CAX*_*3*_*, CCR, COPT*_*5*_*, DR, GAUT, GSR, GST, HMA*_*3*_, and* SOD*	Yin et al. (2022)
Mulberry (*Morus cultivars* L.)	Cd (20 μM; 7 d)	Roots	• Cd exposure negatively impacted genes maintaining cell wall organization, with major downregulation of genes for cell division • Comparative transcriptomic analysis revealed that the G12 cultivar had higher expression of genes for Cd-chelation and transporter genes (*AGT2, MT2, HIPP26, MTP9* and *DTX43/44*) than the F12 cultivar, while F12 had higher expression of *PCR2* and *ABCC2*	Fan et al. (2022)
Benth (*Nicotiana benthamiana* L.)	Zn (40, 440, and 1100 µ mL^−1^; 3 d)	Leaves	• Zn exposure induced 7575 DEGs relating to phosphatidylinositol signaling and inositol-phosphate metabolism • Ethylene-responsive transcription factors recorded in abundance	Wang et al. (2022c)
Maize (*Zea mays* L.)	B (20 ppm; 96 h) and NaCl (150 mM; 3 h)	Leaves and roots	• Expression of transcription factors, including *NAC*, *HSF*, *bHLH*, *HD-ZIP*, and *MYB* was observed as a means to manage hormonal imbalances, ROS production, and reduce cell damage, promoting B tolerance	Barua et al. (2022)
Silvergrass (*Miscanthus lutarioriparius* L.)	Pb (150 mg kg^−1^)	Leaves and roots	• 19,332 DEGs identified, regulating photosynthesis and metal ion transporters, phenylpropanoid biosynthesis and metabolism, and secondary metabolite biosynthesis	Wang et al. (2022b)
Soybean (*G. max* L.)	Cd (60 mmol L^−1^; 10 d)	Leaves	• 7541 and 7076 DEGs were identified from two groups • Upregulation of genes encoding phytochelatins synthase, MTPs, NRAMP, and vacuoles protein storage played key roles in Cd transportation and ordering • Plant hormone signal transduction, MAPK signaling pathway, plant starch and sucrose metabolism, and photosynthesis were the most highly enriched pathways	Gong et al. (2023)
Wheat (*Triticum aestivum* L.)	Cd (10 μM; 12 d)	Leaves and roots	• 20,095 DEGs were identified, including 12,207 in leaves and 10,350 in roots • The most enriched pathways were secondary metabolite biosynthesis, starch and sucrose metabolism, carbohydrate metabolism, and MAPK signaling • Different DEGs involved in photosynthesis, ROS/antioxidant metabolism, MAPK signaling pathway, and element transporters regulated Cd tolerance	Liu et al. (2023a)
Rice (*Oryza sativa* L.)	As-III (1 mg L^−1^; 8 d)	Leaves and roots	• 4195 and 1842 DEGs were identified in root and leaves, respectively • Most DEGs were involved in diverse pathways like carotenoid biosynthesis, MAPK signaling pathway, aminoacyl-tRNA biosynthesis, carbon fixation in photosynthetic organisms, and alanine, aspartate and glutamate metabolisms • 505 DEGs from TF families were also identified, including MYB, AP2/ERF, WRKY, NAC, bHLH, and C2H2, suggesting their role in As tolerance	Xu et al. (2023)
Rice (*O. sativa* L.)	As (10 and 50 µM L^−1^)	Roots	• A total of 3860 novel genes were identified and enriched in different classes • Diverse transcription factors were expressed including WRKY, FAR1, NAC, bZIP, bHLH, B3, C2H2, ERF, M-type-MADS, MIKC-MADS, MYB- and –related • Most of the DEGs were associated with heat shock and stress responses, detoxification, transporters and metals-related, and phytohormones	Sehar et al. (2023)
Rice (*O. sativa* L.)	Hg (20 µmol L^−1^ HgCl_2_) and Hg plus elemental S (100 mg L^−1^; 3 d)	Roots	• A total of 3411, 2730, and 581 DEGs were identified in CK vs. Hg, CK vs. Hg + S, and Hg vs. Hg + S datasets, respectively • Most enriched pathways were biosynthesis and metabolism, expression regulation, transport, stimulus–response, oxidation–reduction, and cell wall biogenesis • Most of the biological process-associated genes were upregulated against Hg as compared to CK, but downregulated in the Hg + S treatment	Huang et al. (2024)
Wheat (*T. aestivum* L.)	In(NO_3_)_3_ (10, 30, 50, and 100 μM; 24 h)	Roots	• A total of 4439 DEGs were discovered in roots, including 2478 upregulated and 1961 downregulated • Indium affected the expression of several genes associated with cell wall composition and metabolism	Qian et al. (2024)
Apple (*Malus* spp.)	Se (3, 6, 9, 12, 24, and 48 μM; 28 d)	Roots	• A total of 993 (624 upregulated and 369 downregulated), 2595 (1032 upregulated and 1563 downregulated), and 5090 (2425 upregulated and 2665 downregulated) DEGs were discovered in the different pairwise comparisons • Most of the DEGs were enriched in cell wall biogenesis, oxidoreductase activity, anchored component of membrane, and supramolecular polymer to enhance Se tolerance • Most enriched pathways were nitrogen metabolism, fatty acid degradation, tyrosine metabolism, phenylpropanoid biosynthesis, cyanoamino acid metabolism, biosynthesis of secondary metabolites, galactose metabolism, fructose and mannose metabolism, amino acid and organic acid metabolism contributing to Se tolerance	Liu et al. (2024)

Note: Abbreviations are explained in the main text

White clover (*Trifolium repens*) exposed to Cd showed differential gene expression related to glutathione metabolism and phenylpropanoid lignin biosynthesis in roots, photosynthesis in leaves, and transferase activity, oxidoreductase activity, and abscisic acid (ABA) signal transduction in shoots (Wu et al. 2022b). Similarly, Cd-exposed crown flower (*Calotropis gigantea*) plants showed differential expression of genes related to oxidative stress in roots, and various Cd transport mechanisms like absorption, efflux or compartmentalization, cell wall structuring, antioxidation, and chelation in leaves (Yang et al. 2022a).

Beneficial elements are not vital for all crops but may be essential for specific plant taxa and could enhance plant growth and yield exposed to metal(loid) toxicity (Awasthi et al. 2022; Nunes da Silva et al. 2022). For instance, silicon (Si) can reportedly reduce metal(loid) toxicity in various species (Huang et al. 2021; Shen et al. 2021b; Sun et al. 2022; Zhou et al. 2022). Transcriptomic analysis revealed that Si treatment reversed the expression of various genes in Cd- and As-exposed rice plants, returning them to control levels (Huang et al. 2021). Similarly, Cd-stressed rice plants treated with foliar Si increased the expression of genes related to nutrient transport, ABC transporters, bivalent cation-transporters, carbohydrate and secondary metabolite biosynthesis, and cytochrome oxidase activity (Sun et al. 2022). Furthermore, Si-alleviated Cd stress in soybean by regulating differentially expressed genes (DEGs) for ABC transporters, ZIP transporters, *NRAMP* transporters, and various genes, including *XTH*, *PME/PMEI*, *GST*, and *PRX* (Zhou et al. 2022). Moreover, sulfur (S)-mediated Cd stress in spinach by increasing the expression of genes related to various transmembrane exporters, including cation/proton exchanger-3 (CAX3), *NRAMP* transporters-specifically *NARMP5*, ABC transporters, glutathione, and cysteine (Shen et al. 2021b).

Significant downregulation in DEGs has been reported in rice exposed to As(III) and As(V) (Di et al. 2021; Peña-Garcia et al. 2021; Khare et al. 2022; Meselhy et al. 2021). The *Arsenic Stress-Related F-Box* (*ASRF*) gene was recognized as a prominent factor in controlling As(V) responses in *A. thaliana*, regulating phosphate and cellular homeostasis (Peña-Garcia et al. 2021). This study reported the upregulation of 63 transporter genes related to ABC transporters, metabolite transporters, amino acid transporters, proton transmembrane transporters, sugar, metals/metalloids, cation, cyclic nucleotide-gated channel protein genes, peptide transporters, MATE-efflux family protein genes, and glucose 6-phosphate/phosphate translocator 1 (GPT1) (Peña-Garcia et al. 2021). Like Cd, transcriptomic analysis revealed a high number of unique DEGs in *A. thaliana* under combined stress of limited S and As stress, with phytochelatins, glutathione, and cysteine as the major players disrupting As toxicity (Khare et al. 2022). In another study, Meselhy et al. (2021) reported a size-dependent ability of nano-sized S to alleviate As toxicity in rice.

Zucchini (*Cucurbita pepo*) exposed to copper (Cu) oxide nanoparticles (CuO NPs) produced 4420 DEGs in roots and 3122 in shoots, while copper oxide (CuO bulk) and copper sulfate (CuSO_4_) produced 9924 and 9103 DEGs in roots and 6540 and 4747 in shoots, respectively (Marmiroli et al. 2021). The roots of cogon grass (*Imperata cylindrica*) after Cu exposure produced 7386 DEGs, with 3558 upregulated and 3828 downregulated (Vidal et al. 2021). In the same study, the shoots only produced 36 DEGs, with 13 upregulated and 23 downregulated. The authors proposed that the cytoskeleton acted as a Cu binding mechanism in roots, leading to Cu tolerance (Vidal et al. 2021). Furthermore, Cu exposure upregulated 715 and downregulated 573 DEGs in pomelo (*Citrus grandis*) leaves, with upregulated genes related to antioxidant activity, oxidation–reduction homeostasis, thermal energy dissipation, and photorespiration activities (Wu et al. 2021a). The authors also reported changes in gene expression patterns involved in cell wall metabolism and leaf growth (Wu et al. 2021a). 5-Aminolevulinic acid (ALA) reduced Cu stress in grapevine (*Vitis vinifera* L.) by altering the expression of *ALAD*, *CHLH*, *DHAR*, and *RCA* genes related to absorption, transport, photosynthesis, Chl metabolism, and antioxidation (Yang et al. 2022b).

Chinese silver grass (*Miscanthus sinensis*) exposed to Cr toxicity produced 83,645 DEGs related to metal(loid) transport, metal(loid) ion chelation, photosynthesis, ABC transporters, and glutathione metabolism (Nie et al. 2021). Another study identified 1060 DEGs in radish (*Raphanus sativus* L.) exposed to Cd toxicity (623 upregulated and 437 downregulated) related to the regulation of cellular components and responses, oxidative responses, auxin signaling pathways, secondary metabolism, and aromatic compound biosynthesis (Peng et al. 2021).

Transcriptomic analysis of red clover (*Trifolium pratense*) exposed to varying Pb concentrations (500, 1000, 2000, and 3000 mg kg^–1^) identified 65 (28 upregulated and 37 downregulated), 311 (105 upregulated and 206 downregulated), 1288 (935 upregulated and 253 downregulated), and 596 (216 upregulated and 380 downregulated) DEGs, respectively (Meng et al. 2022). In another study, Liu et al. (2022) carried out transcriptome analysis of common green branched weed (*Cladophora rupestris*) exposed to 5 mg L^–1^ Pb produced 3783 upregulated and 4312 downregulated DEGs related to various ABC transporters, glutathione metabolism, phenylpropanoid biosynthesis, mitogen-activated protein kinase, and other hormone signaling pathways (Liu et al. 2022), suggesting their key role in Pb tolerance. A recent transcriptomic analysis of pearl millet (*Pennisetum glaucum* L.) exposed to Fe and Zn toxicity identified 406 DEGs at the panicle initiation stage, 349 at the flowering stage, and 378 at the milking stage related to various metabolic processes, including Fe and Zn uptake and transport, catalytic activity, transferase activity, metal/metalloid ion binding, ATP binding, DNA binding, heme binding, oxidoreductase activity, peroxidase activity, and nutrient reservoir activity (Satyavathi et al. 2022).

In summary, transcriptomics provides valuable insights into the molecular mechanisms underlying plant responses to metal(loid) toxicity, which can be harnessed to improve crop tolerance to metal(loid) stress, thereby contributing to global food security efforts.

### Proteomic innovations: deciphering cellular responses to improve metal(loid)s tolerance

As previously mentioned, molecular mechanisms at the genomic level play an important role in plant tolerance to metal(loid)s but are not always reflected at the protein level (Millán-Zambrano et al. 2022). Detailed studies on translational and post-translational modifications are crucial for identifying target proteins engaging in metal(loid)-induced responses (Muleya et al. 2022). Proteomic analyses quantify the entire protein complement expressed by a genome and can be performed from the bottom-up or top-down, providing large-scale data on plant system changes and regulations (Cassidy et al. 2021). From an operational perspective, proteomics has evolved rapidly from the earliest generations to include (1) 2DE-MS, (2) isobaric/isotopic tagging, (3) shotgun and gel/label-free approaches, and (4) mass western, targeted, SRM/MRM methodologies (Jorrin-Novo et al. 2019). As a result, markers involved in metal(loid) tolerance have been identified in the proteomes of numerous metal(loid)-stressed plants (Table 2) (Zou et al. 2021).

**Table 2:** Summary of some recent proteomic studies identifying key players associated with metal(loid) tolerance in different plant species

Plant species	Stress condition	Tissue	Key findings	Reference
Tobacco (*Nicotiana tabacum* L.)	Cd (100 μmol L^−1^) and Zn (200 μmol L^−1^; 10 d)	Leaves	• Compared to Zn, Cd stress severely inhibited photosynthesis and Chl synthesis-related proteins, Fd-dependent nitrogen metabolism, and ROS scavenging	Zhang et al. (2020)
Wheat (*Triticum aestivum* L.)	Cd (50 μM; 24 h)	Roots	• DNA replication and repair, protein metabolism, and glutathione metabolism-related proteins were differentially expressed	Jian et al. (2020)
Barrel clover (*Medicago truncatula* L.)	Cd^2+^, Co^2+^, Mn^2+^, Zn^2+^, and Fe^2+^ (24 h)	Leaves, stems, and roots	• Identified 12 MTPs grouped in three major cation diffusion facilitators (CDFs): Mn-CDFs, Zn-CDFs, and Fe/Zn-CDFs	El-Sappah et al. (2021)
Soybean (*Glycine max* L.)	Mn (100 μM; 7 d)	Shoots and roots	• Identified GmMTP protein, as an ER-localized Mn transporter, helping to overcome Mn toxicity by stimulating Mn exportation and increasing sequestration into intracellular compartments	Li et al. (2021)
Common reed (*Phragmites australis* L.)	Cu (0.5, 1.0, 2.5, 5.0, and 10.0 mg L^–1^; 21 d)	Buds	• Inhibition of photosynthesis by downregulation of PSI, PSII, and LHCII • Changes in the antioxidant component pool, including ascorbic acid and proline	Wu et al. (2021b)
Maize (*Zea mays* L.)	Cr (100 µM; 7 d)	Roots	• Increased hydrogen peroxide and lipid peroxidation, glutathione peroxidase, and superoxide dismutase under Cr exposure	Terzi and Yıldız (2021)
Black cottonwood (*Populus trichocarpa* L.)	Pb (0.75 µm; 24 h)	Leaves and roots	• Identified proteins with increased abundance involved in lignin and flavonoid biosynthesis pathway • Upregulated cell wall metabolism-related proteins such as xyloglucan • Pb induced post-transcriptional regulation	Shen et al. (2021a)
Rapeseed (*Brassica napus* L.)	As (200 μmol L^−1^; 7 d)	Leaves	• DEPs related to ribosomes and secondary metabolism biosynthesis • As-responsive proteins included those involved in primary metabolism, oxidative stress, and defense systems	Farooq et al. (2021)
Black nightshade (*Solanum nigrum* L.)	Cd (25 and 100 µm; 3 weeks)	Leaves	• Identified 105 DEPs • Under low-Cd dose, 47 DEPs primarily involved in primary metabolic activities • Under high Cd dose, 92 DEPs primarily involved in photosynthesis, energy metabolism, production of ROS, and phytochelatins	Song et al. (2023)
Castor bean (*Ricinus communis* L.)	Cd (300, 700, and 1000 mg L^−1^; 21 d)	Roots	• Significantly upregulated DEPs involved in defense, detoxification, and energy metabolism • Significantly upregulated plasma membrane ATPase encoding gene (*RcHA4*) • In response to Cd treatment, castor plants improved cell wall strength and stimulated programmed cell death to inhibit Cd^2+^ absorption by root systems	Huibo et al. (2023)
Wheat (*T. aestivum* L.)	Cd (15 µm; 14 d)	Roots	• Among different groupwise comparisons, DEPs were mostly enriched in lipid metabolism, unsaturated fatty acid biosynthesis, flavonoid biosynthesis, glutathione metabolism, alpha-linolenic acid metabolism, phenylpropanoid biosynthesis, cysteine and methionine metabolism, and starch and sucrose metabolism pathways • Enhanced Cd tolerance related to increased antioxidant activities, plasma membrane stability, nitrogen metabolism, and endoplasmic reticulum homeostasis	Zhang et al. (2023)
Rapeseed (*B. napus* L.)	Cr (10, 50, and 100 μM; 7 d)	Seedlings	• A total of 60 proteins were greatly altered by Cr treatment, and 54 proteins were discovered by MS • Cr tolerance could be increased by triggering photosynthetic efficiency, ROS scavenging ability, protein biosynthesis and processing, and other adaptive responses	Doğuş et al. (2023)
Chinese cabbage (*B. campestris* L.)	Cd (200 μM; 24 h)	Roots	• A total of 1514 DEPs were discovered, including 451 upregulated and 973 downregulated • Significantly enriched pathways include different metabolisms such as cysteine and methionine, phenylalanine, tyrosine, and plant–pathogen interaction, plant hormone signal transduction, ribosome biogenesis in eukaryotes, phosphatidylinositol signaling system, protein export, RNA polymerase, isoquinoline alkaloid biosynthesis, and flavone and flavonol biosynthesis	Sun et al. (2023)
Rice (*Oryza sativa* L.)	Cd (5 and 10 μM; 14 d)	Roots	• A total of 9733 quantified proteins were detected • 3945 DEPs (1906 upregulated and 2039 downregulated), and 2844 DEPs (1103 upregulated and 1741 downregulated) were identified in two comparisons • Significantly upregulated DEPs include those associated with antioxidation and ROS scavenging, peroxidases, aldehyde dehydrogenase, and cell wall modification	Kuang et al. (2024)

Note: Abbreviations are explained in the main text

For instance, quantitative succinyl-proteome profiling using LC–MS-based proteomics in turnip (*Brassica rapa* L.) seedlings treated with Cd (20 µM) identified 547 succinylated sites on 256 proteins, with statistically changed intensities for nine succinylation sites on eight proteins (Li et al. 2022d). Interestingly, these differentially succinylated sites were located in some oxidative proteins, including glycolate oxidase, catalase, and glutathione S-transferase (Li et al. 2022d). In another study, sub-organelle proteomics of Indian mustard (*Brassica juncea* L.) under Cd stress identified the regulation of specific defense and signaling pathways (Sehrawat and Deswal 2022).

Genome-wide proteomics analysis revealed different transmembrane transporters such as cation diffusion facilitators (CDF), metal transport proteins (MTPs), and zinc–iron permease involved in the transportation of metal(loid)s to intracellular organelles (Tiwari and Lata 2022). The CDFs were involved in the homeostasis of divalent metal cations such as Zn, Mn, Cd, and Co, transporting excess ions into vacuoles (Tiwari and Lata 2022). A recent genome-wide analysis identified 12 CDFs in barrel clover (*Medicago truncatula* L.) under Cd, Zn, Mn, and Fe toxicity, with RNA-seq and gene ontology revealing their potential role in plant growth and development (El-Sappah et al. 2021). Likewise, the expression of *BjCET1* (an MTP) significantly increased in Indian mustard exposed to Cd and Zn, increasing metal(loid) tolerance by reducing Cd and Zn accumulation (Han et al. 2022). Another recent genome-wide study identified 24 *AhMTP* proteins in peanut (*Arachis hypogea* L.) belonging to three substrate-specific clusters of Zn-CDFs, Zn/Fe-CDFs, and Mn-CDFs, preferentially expressed in generative plant parts, suggesting their involvement in metal transport during seed development (Wang et al. 2022e).

Most proteins functionally interact with other small and large molecules (including other proteins and metabolites) to maintain cellular homeostasis. Protein–metabolite interactions are vital in cell signaling pathways (Venegas-Molina et al. 2023). Large-scale proteomics analyses can unravel the networking between proteins and metabolic pathways (Yusuf et al. 2022), but studies are limited. Recent developments include chemoproteomic workflows and an interactomics method using nuclear magnetic resonance (NMR) to systematically identify targeted metabolite–protein interactions (Li et al. 2022c). Techniques like limited proteolysis-coupled mass spectrometry (LiP-MS) have also been introduced to identify novel protein–metabolite interactions related to plant regulatory mechanisms (Venegas-Molina et al. 2023).

An isobaric tags for relative and absolute quantitation (iTRAQ)-based technique was recently used to identify differentially expressed proteins (DEPs) and key metabolic pathways in tobacco (*Nicotiana tabacum* L.) plants exposed to Cu stress, with 180 DEPs identified (78 upregulated and 102 downregulated) (Gao et al. 2022). Further analysis functionally categorized these DEPs into 65 classes related to carbon metabolism, glycolysis/gluconeogenesis pathways, and secondary metabolite metabolism, with peroxidase 7 among the most significant upregulated DEPs attributed to enhanced Cu tolerance (Gao et al. 2022). In another study, iTRAQ was used to unravel the mechanism of Cd hyperaccumulation by comparing DEPs for Cd and Zn in black nightshade (*Solanum nigrum*), revealing that protein export, ribosome, amino sugar, and nucleotide sugar metabolism mainly contribute to Cd accumulation (Dai et al. 2022). A similar study using iTRAQ-based proteomics explored the Cd hyperaccumulation mechanism in *S. nigrum* by comparing DEPs associated with Cu accumulation (non-Cu hyperaccumulator), revealing 27 co-interesting DEPs involved in metabolic pathways, which might have resulted in Cd enrichment and translocation factors > 1 (Jia et al. 2022).

Label-free shotgun proteomics analysis in crowngrass (*Paspalum fasciculatum*) leaves exposed to Cd stress identified 329 variable proteins closely related to carbon metabolism, protein metabolism, photosynthesis, plant defensive system, and signaling pathways (Salas-Moreno et al. 2022b). In the same study, quantitative proteomics analysis showed that proteins like Ras-related protein RABA1e, heat shock cognate 70 kDa protein 2, actin-7, and actin-1 are involved in the Cd-induced tolerance response (Salas-Moreno et al. 2022b). Similarly, an LC–MS/MS-based study on crowngrass under Pb stress identified 323 proteins, mainly involved in primary metabolism and antioxidant defenses, that coordinated an improved physiological response to Pb (Salas-Moreno et al. 2022a). Another recent study in rice roots reported that DEPs under both Fe deficiency and excess were commonly associated with carbon and amino acid metabolism, antioxidant apparatus, and localization (Zhang et al. 2022c). However, further interpretation revealed that proteins related to ribosome and endocytosis were specifically regulated under excess Fe, while phenylpropanoid, cysteine, and methionine-related proteins were regulated under Fe deficiency (Zhang et al. 2022c).

A gel-based proteomic study showed that 34 proteins, mainly involved in photosynthesis and energy metabolism, were remarkably regulated by nitric oxide Cd-treated barley (*Hordeum vulgare* L.) (Alp et al. 2022). Furthermore, a quantitative proteome profile of rapeseed (*Brassica napus* L.) under Cr stress revealed downregulated proteins, including glycine-rich RNA-binding protein, lactoylglutathione lyase, fructose-bisphosphate aldolase, and glutamine synthetase, with Cys application alleviating these effects and adjusting the regulation of some upregulated proteins under Cr stress, including those related to oxidative defense, energy, and amino acid metabolisms, leading to Cr tolerance (Yıldız and Terzi 2021).

In another study, proteomics analysis of lettuce (*Lactuca sativa* L.) under Cd stress (20 µM) revealed downregulated expression of photosynthetic machinery-related proteins, with fulvic acid application remarkably upregulating the expression of light-harvesting proteins, reaction center, and electron transport-related proteins, increasing S metabolism, and restoring redox homeostasis, mitigating Cd toxicity (Chen et al. 2022). In a study, Zn application reduced *Salvia sclarea* plant Cd accumulation, with proteomic analysis identifying membrane proteins responsible for the modulation of Zn/Cd transport (Sperdouli et al. 2022). iTRAQ and parallel reaction monitoring (PRM)-based quantitative proteomics identified 4008 proteins in rice plants under Cd stress, with 332 DEPs functionally involved in glutathione metabolism and phenylpropanoid biosynthesis, and ion-transport-related DEPs engaged in transmembrane transport regulation, improving Cd tolerance (Zhang et al. 2022a).

In summary, proteomics offers a deeper understanding of how plants respond to metal(loid) stress at the protein level. Advances in proteomics techniques have enabled researchers to identify key proteins and processes involved in metal(loid) tolerance, offering insights that can be applied to improve plant resilience to metal(loid) toxicity.

### Metabolomics insights into metal(loid)s tolerance: pathways to biochemical dynamics

Metabolomics, the study of “measuring quantitative and qualitative metabolite levels” of stressed plant/tissue/organelle/single cell, is a branch of systems biology that is used to investigate stress-responsive pathways in plants (Jamla et al. 2021; Raza 2022; Raza et al. 2022; Lanekoff et al. 2022). Metabolomics deals with metabolite changes, data mining, and bioinformatics analysis, and there are several analytical techniques used to identify key metabolites and metabolic pathways in plants (Fig. 1) (Jamla et al. 2021; Raza 2022; Raza et al. 2022; Lanekoff et al. 2022). Plants typically secrete primary (amino acids, alcohols, vitamins, polyols, organic acids, and nucleotides) and secondary (tocopherols, inositols) metabolites as end products or intermediates in different pathways, which could be explored via metabolic engineering (Irfan et al. 2021). During metal(loid) stress, the number and type of plant metabolites fluctuate (Table 3). This section identifies key findings from recent metabolomics studies on plants exposed to metal(loid) toxicity.

**Table 3:** Some recent examples of metal(loid)-mediated metabolomics studies in different plant species

Plant species	Stress conditions	Tissue used	Analytical platform	Key observations	Reference
Rice (*Oryza sativa* L.)	Fe (15 mM FeSO_4_; 2 d)	Roots and shoot	UPLC-MS	• Overall, 1022 and 707 metabolites found in shoots and roots, respectively • Downregulation of organic acids like oxoglutaric, ketoglutaramic, and succinic acid • Secondary metabolites, including flavanones and amino acids, were highly regulated, highlighting their antioxidative properties for Fe toxicity	Kar et al. (2022)
Rice (*O. sativa* L.)	As and Fe (142.5 mg kg^–1^)	Roots and straw	LC–MS/MS	• Detected 448 metabolites, with metabolites related to lipid metabolism upregulated • Iron-oxidizing bacteria reduced As content by 6.25–12.31% upon treatment • FeOB holds great potential to remediate As toxicity in soil, enhancing soil resistance soil to peroxide (As pollution)	Qian et al. (2022)
Rice (*O. sativa* L.)	As (1 and 1000 μM)	Roots and aerial parts	LC–MS	• Forty metabolites identified • Five major metabolic pathways altered: aminoacyl-tRNA biosynthesis, glycine, serine, and threonine metabolism, arginine and proline metabolism, and arginine biosynthesis	Pérez-Cova et al. (2022)
Tobacco (*Nicotiana tabacum* L.)	Cd (5 μM CdCl_2_; 5 d)	Leaves and roots	UHPLC	• About 150 and 76 metabolites accumulated in roots and leaves, respectively • Biosynthesis of nicotinate and flavonol, nicotinamide metabolism, arginine and proline metabolism • Foliar application of Zn and Fe on Cd-stressed plants alleviated plant growth by reprogramming metabolome profile	Zou et al. (2022)
Pomelo (*Citrus sinensis* L.)	Cu (300 μM; 7 d)	Leaves	LC–MS	• Identified 502 metabolites, including lipids, nucleotides, alkaloids, flavonoids, phenolic acids, tannins, terpenoids, and quinones • Downregulation of phospholipids and upregulation of Trp metabolism • Increased pH decreased Cu toxicity and its effects on carbohydrate, lipid, and amino acid metabolism	Zhang et al. (2022b)
Red clover (*Trifolium pratense* L.)	Pb (0, 500, 1000, 2000, and 3000 mg kg^–1^)	Stem	UPLC, MS/MS, and QTRAP	• Nine hundred and fivemetabolites identified • Pb3000 group had increased lipid, vitamin, and phenolic acid levels • Pb500 group had higher nucleotide, flavonoid, vitamin, organic acid, and amino acid levels than the Pb 3000 group, but lipid, terpene, and phenolic acid levels decreased	Meng et al. (2022)
Rice (*O. sativa* L.)	Pb (0, 50, and 100 µmol L^−1^; 10 d)	Leaves	GC-TOF/MS	• Significantly accumulated 44 metabolites, comprising sugars, polyols, amino acids, organic acids, and fatty acids • Sugars, organic acids, amino acids, and secondary metabolites increased, improving antioxidant capacity • Pb stress enhanced glactose metabolism and sugar metabolism (starch and sucrose)	Wang et al. (2023)
Rice (*O. sativa* L.)	Cd (30 mg kg^−1^; 40 d)	Leaves	UHPLC–MS/MS	• Sixteen differential metabolites were observed in the Cd treatment • Differential metabolites (such as chrysin and galangin) verified the disturbance of flavonoid biosynthesis in response to Cd treatment	Qiang et al. (2023)
Rice (*O. sativa* L.)	Cd (0, 25, 50, and 100 µmol L^−1^; 5 d)	Roots and shoots	GC/MS	• Cd-triggered variations in metabolite accumulation, including amino acids, organic acids, sugars and their derivatives, phenolics, nitrogen bases, and purine metabolites • Cd treatment affected primary and secondary metabolism pathways	Paul and Das (2023)
Rice (*O. sativa* L.)	Cu^2+^ (100 μM; 10 d)	Roots	LC–MS/MS	• Six hundred and ninety five metabolites were identified, including 23 were upregulated and 297 were downregulated • Differential metabolites include carboxylic acids and derivatives, benzene and substituted derivatives, carbonyl compounds, cinnamic acids and derivatives, fatty acyls and organ nitrogen compounds • Highly enriched pathways include TCA cycle, purine, and starch and sucrose metabolisms	Cao et al. (2023)
Rice (*O. sativa* L.)	As(III) (2 and 4 mg L^−1^)	Stems	UPLC-MS	• Nine hundred and eighteen significant DAMs were identified • Pathways linked to plant growth, development and stress tolerance were highly enriched • The dermatan l-iduronate DEM was the key player separating metabolites in As(III)-treated to CK group	Ma et al. (2023)
Wheat (*Triticum aestivum* L.)	In(NO_3_)_3_ (10, 30, 50, and 100 μM; 24 h)	Roots	LC–MS/MS	• Key metabolites such as cinnamic acid, p-coumaraldehyde, caffeic acid, ferulic acid and coniferyl aldehyde were accumulated in roots after In(NO_3_)_3_ treatment • Indium significantly altered different metabolism such as secondary, amino acid, lipid, carbohydrate, energy, nucleotide, and other metabolic pathways • Phenylpropanoid and benzoxazinoid biosynthesis pathways were highly upregulated	Qian et al. (2024)
Apple (*Malus* spp.)	Se (3, 6, 9, 12, 24, and 48 μM; 28 d)	Roots	LC–MS	• A total of 1243 metabolites were noticed in all comparisons, which were classified into 11 subclasses • Forty five common DAMs were discovered between the three comparisons • Highly accumulated DAMs were phenolic acids, organic acids, terpenoids and alkaloids, and amino acids and their derivatives and reduced accumulation of lipids • Top-enriched pathways include biosynthesis of isoquinoline alkaloid, flavone and flavonol, valine, leucine, and isoleucine, and metabolism of ascorbate and aldarate, pentose and glucuronate, tryptophan, phenylalanine, fructose and mannose, galactose, arginine and proline, and citrate cycle	Liu et al. (2024)

Note: Abbreviations are explained in the main text

A metabolomics analysis of miswak (*Salvadora persica*) plants exposed to As toxicity and salt stress revealed severe effects on photosynthetic attributes and stomatal regulation (Patel and Parida 2022). The study identified 64 differentially accumulated metabolites (DAMs) under As stress, including primary metabolites (polyphenols, amino acids, citrate cycle intermediates) and many phytohormones. Moreover, salt addition alleviated disturbed metabolic pathways, including the citrate cycle and glyoxylate, dicarboxylate, amino acid, and glutathione metabolisms (Patel and Parida 2022). Chickpea (*Cicer arietinum* L.) plants exposed to As(V) toxicity had disrupted plant metabolism, including carbohydrate-metabolizing enzyme and antioxidative activities (Adhikary et al. 2022). However, treatment with plant growth-promoting bacteria (PGPB; *Pseudomonas citronellolis*) mitigated these effects and helped maintain cellular homeostasis. The metabolomics analysis identified 48 metabolites involved in metabolic pathways, including carbohydrates, amino acids, and fatty acids (Adhikary et al. 2022).

A comparative metabolomics study of wheat (*Triticum aestivum* sp. *tritici*) cultivars under Al stress identified 100 significantly variable metabolites, including phenolic compounds (flavonoids glycosides and hydroxycinnamic acid), organic acids, fatty acids, and amino acids (Mashabela et al. 2022). Metabolic adaptations were observed in orange trees (*Citrus sinensis* L.) under Al toxicity, particularly at higher pH levels (Wu et al. 2022a). These adaptations helped maintain phosphate and energy homeostasis, scavenge ROS and some aldehydes, and accumulate secondary metabolites, such as phenol amides, polyphenols, and proanthocyanidines, to counter Al stress (Wu et al. 2022a).

Metabolomic analysis of common reed (*Phragmites australis*) exposed to Cu stress revealed the accumulation of ayarin and arginine, which were associated with enhanced resistance to Cu toxicity (Wu et al. 2022c). Amino acid and flavonoid accumulation contributed to antioxidant activity and improved phytoremediation efficiency (Wu et al. 2022c). A metabolomic study in Chinese brake fern (*Pteris vittata* L.) exposed to various metal(loid)s, including Zn, Pb, Sb, Ag, Ni, and Mn, identified 359 metabolites in aerial and subterranean samples (Nguyen et al. 2022). Flavonoid patterns induced by Pb, Ni, and Ag were associated with defense mechanisms in different tissues. Major amino acids (glutamic acid, argininosuccinic acid, threonic acid, pyroglutamic acid, and lysine) induced by As toxicity were also identified, potentially providing insights for reprogramming metabolites to induce tolerance against metal(loid)s (Nguyen et al. 2022). Metabolite fingerprinting revealed substantial alterations in 24 metabolites in medicinal leech (*Whitmania pigra*) exposed to varying levels of Pb stress, some of which were dose-dependent (Luo et al. 2020). These metabolites included lipids, nucleotides, and dipeptides involved in metabolic pathways such as glycerophospholipid metabolism, sphingolipid metabolism, and nucleotide metabolism (Luo et al. 2020).

Metabolomics analysis of Chinese cabbage (*Brassica rapa* L.) exposed to different levels of Cd stress identified 2275 and 903 metabolites in the positive ion (ESI +) and negative ion (ESI–) detection models, with 18 DAMs highlighted (He et al. 2022). The study highlighted the role of glutathione (GSH) metabolism in reducing Cd accumulation (He et al. 2022). In another study, B application inhibited Cd uptake in wheat, with the metabolomic analysis identifying 198, 680, and 204 DAMs under different Cd treatments involved in linoleic acid metabolism, glycolysis, and sphingolipid metabolism (Wang et al. 2022e).

Metabolomic profiling of lettuce leaves under Cd stress revealed reductions in two amino acids (serine and l-isoleucine), increased glutamic acid, and disrupted methyl maleic acid activity and glyoxylate and dicarboxylate metabolisms (Zeb et al. 2022). Metabolomic profiling of *S. nigrum* under Cd stress revealed seven significantly affected metabolism pathways (carbohydrates, amino acids, and nucleotides, including glutamic acid, pyruvic acid, cytidine and uridine, D-fructose and beta-alanine) and 19 DAMs (Wang et al. 2022a). Metabolomic analysis of muskmelon (*Cucumis melo* L.) exposed to Cd stress identified 247 DAMs (222 upregulated and 25 downregulated), primarily related to flavonoid and jasmonic acid (JA) biosynthesis (Gao et al. 2022). Foliar spraying of Fe_3_O_4_ and ZnO mitigated the adverse effects of Cd toxicity on tobacco plant growth, with the metabolomics analysis identifying 150 and 76 metabolites in roots and leaves, respectively, mainly involved in arginine and proline metabolism, nicotinate, and flavanol and amino acid biosynthesis (Zou et al. 2022).

In summary, metabolomics offers valuable insights into the metabolic adaptations and responses of plants exposed to metal(loid) stress. Various studies have identified specific metabolites and metabolic pathways that play crucial roles in metal(loid) tolerance mechanisms and offer potential strategies for improving plant resilience to such stress conditions.

### Insights from the integration of different omics approaches

Integrating various omics approaches, including transcriptomics, proteomics, and metabolomics, in a single experiment can offer new insights into the complex molecular mechanisms governing metal(loid) tolerance in plants. This integrated approach permits the discovery of key genes, proteins, and metabolites and associated pathways involved in metal(loid) stress responses and tolerance (Figs. 1 and 3). The data obtained from integrated approaches can be used to advance crops with increased metal(loid) tolerance, yield, and other anticipated agronomic traits. For instance, a recent integrated transcriptomics and metabolomics analysis revealed the molecular mechanisms underlying melatonin-mediated Cd detoxification (Li et al. 2022b). The study identified several DEGs and DAMs related to valine, leucine, and isoleucine degradation, ABC transporters, and alpha-linolenic acid metabolism. Three major mechanisms—(1) increased antioxidant capacity, (2) secondary metabolite accumulation, and (3) regulated ion transportation—were involved in MT-mediated Cd detoxification (Li et al. 2022b). An earlier study combining metabolomics and transcriptomics also identified these three mechanisms involved in nitric oxide-mediated Cd detoxification (Zhu et al. 2020). An untargeted metabolomics analysis in wheat grown under Cd and Pb stress showed that *Enterobacter bugandensis* TJ6 (a metal-immobilizing bacterium) triggered the synthesis of indole-3-acetic acid, betaine, and arginine metabolites (Han et al. 2021). Furthermore, label-free proteomics identified several proteins involved in protein DNA complexes, DNA packaging complexes, and peroxidase activity among the DEPs (Han et al. 2021).

A combined transcriptome and metabolome analysis was used to investigate the nano TiO_2_ or TiO_2_-Cd tolerance mechanism in rice, identifying 423 DEGs and 16 DAMs under Cd stress, 299 DEGs and 6 DAMs under nano TiO_2_, and 1660 DEGs and 181 DAMs under TiO_2_-Cd (Qiang et al. (2023). Notably, DEGs encoding chalcone isomerase and hydroxycinnamoyl transferase and DAMs like chrysin and galangin disrupted flavonoid biogenesis in Cd-treated plants (Qiang et al. 2023). Likewise, an integrated transcriptome and metabolome analysis revealed that flavonoid biosynthesis pathways play a vital role in regulating Cd toxicity in sorghum (*Sorghum bicolor* L.) roots, with 2683 DEGs and 160 DAMs in Cd-treated sorghum roots (Jiao et al. 2023). An integrated proteomics and metabolomics analysis of castor (*Ricinus communis* L.) plants under Cd toxicity identified highly upregulated DEPs involved in defense and detoxification, energy metabolism, and DAMs like organic acids and flavonoids (Huibo et al. 2023). Functional validation of the plasma membrane ATPase encoding gene (*RcHA4*) in wild-type *A. thaliana* revealed its vital role in enhancing Cd tolerance in castor plants (Huibo et al. 2023).

An integrated transcriptomic and metabolomic analysis investigated the Cd and Mn tolerance mechanisms in the Mn/Cd hyperaccumulator plumed cockscomb (*Celosia argentea* Linn), identifying 3960 DEGs, with several related to metal transport and ATP transporter families (Yu et al. 2023a). Notably, Cd and Mn toxicity upregulated three transporter genes (*HMA3*, *ABCC15*, and *ATPase4*). The 33 DAMs identified under Mn stress and 77 identified under Cd stress were mainly involved in ABC transporter and GSH pathways, which could be vital in metal detoxification (Yu et al. 2023a). Integrated metabolome and transcriptome analysis of water lettuce (*Pistia stratiotes*) exposed to Cd toxicity identified 27 DAMs associated with various metabolic pathways, including unsaturated fatty acids, amino acids (phenylalanine), nucleotides, S compounds, and flavonoids and 3107 DEGs enriched in glutathione metabolism and lignin biosynthesis pathways (Wei et al. 2023).

Integrated omics analysis improves our understanding of the complex molecular mechanisms of metal(loid)s tolerance in diverse plant species. By identifying key genes, proteins, metabolites, and novel or shared metabolic pathways involved in metal(loid) responses and tolerance, this methodology facilitates the development of major crop plants with increased metal(loid) tolerance. Future research should address the challenges associated with data integration and interpretation and validate the functional roles of identified candidates (genes, proteins, and metabolites). The same approach can be applied to manipulate metabolic pathways using metabolic/genetic engineering. In addition, incorporating other omics methods, such as epigenomics and microbiomics, could further enhance our understanding of metal(loid) tolerance in plants.

## Integrating omics data with artificial intelligence and high-throughput phenotyping

Metal(loid) toxicity is a significant challenge in agriculture, and traditional breeding approaches have limitations in developing metal(loid)-tolerant crops. Integrating omics data with artificial intelligence (AI) and HTP presents a promising approach to accelerate and enhance crop breeding for metal(loid) tolerance. Omics methodologies, including genomics, transcriptomics, proteomics, and metabolomics, offer insights into the molecular mechanisms underlying plant responses to metal(loid) toxicity (Fig. 3). These data are invaluable for identifying key genes, proteins, and metabolic pathways involved in tolerance (Esposito et al. 2019; Lakshmi et al. 2021; Jung et al. 2021; Tripodi et al. 2022; Khan et al. 2022; Yan and Wang 2023; Raza et al. 2023).

AI algorithms can be applied to omics data to identify genetic markers associated with metal(loid) tolerance, serving as valuable tools for breeders to select desirable traits efficiently (Harfouche et al. 2019; Yan and Wang 2023). Integrating omics data with AI enables the development of predictive models that can forecast the plant performance under metal(loid) stress, facilitating the identification of promising candidates for further breeding to design elite/superior cultivars (Fig. 3) (Esposito et al. 2019; Lakshmi et al. 2021; Jung et al. 2021; Tripodi et al. 2022; Khan et al. 2022; Yan and Wang 2023).

HTP methods involving automated imaging and sensing technologies enable the rapid collection of plant growth and development data. This information can be integrated with omics and AI approaches to rapidly screen large numbers of plant varieties for their responses to metal(loid) toxicity, facilitating targeted breeding efforts (Lakshmi et al. 2021; Khan et al. 2022).

Machine learning (ML) techniques are used for genomic prediction, selection, and marker-assisted breeding (Harfouche et al. 2019; Esposito et al. 2019; Pazhamala et al. 2021; Varshney et al. 2021; Jung et al. 2021; Khan et al. 2022). Plant breeders use AI tools like ML, deep learning, and predictive analysis to understand plant behaviors under various conditions, including metal(loid) toxicity (Esposito et al. 2019; Yan and Wang 2023; Tripodi et al. 2022). For instance, Yan et al. (2023) used genomic-enabled prediction (GEP) models with ML and linear statistical methods to assess Cd concentration in maize kernels. The authors identified marker density and training populations as key considerations in revealing GEP baseline precision. The GEP models with ridge regression–best linear unbiased prediction performed better than Bayes A and random forest in field trials, with higher GEP precision and lower mean absolute error values. Integrating GEP with GWAS could be a promising strategy for assessing Cd concentration and addressing environmental Cd contamination in maize fields (Yan et al. 2023). Another study integrated ML in GEP models to predict Cd concentrations in crops and detect low-Cd rice cultivars based on microbial taxon-specific resistance mechanisms (Cheng et al. 2023b).

Another study developed a genetic algorithm (GA)-back-propagation neural network to predict Cd concentrations in rice grain based on soil properties (Hou et al. 2018). The predicted Cd concentration could be used to assess human exposure and health risks, enabling timely interventions to reduce Cd transfer in the food chain (Hou et al. 2018). A study in southwestern China reported that Se-rich maize could be grown in Se-poor farmland by studying bioavailable Se levels (Ma et al. 2022). Moreover, Hu et al. (2020) compared different ML models to detect factors influencing the transport of different metal(loid)s (Zn, Cu, Cr, Ni, Hg, Cd, As, and Pb) in soil–crop systems. The random forest model had the best prediction capability, with plant type being the primary controlling factor for all metal(loid)s. The model could predict metal(loid) contents in crops and identify potential control features in metal(loid) bioaccumulation in soil–crop ecosystems (Hu et al. 2020). An artificial neural network (ANN) model was more accurate in forecasting Se bioconcentrations in maize grain than a multivariate linear regression model, permitting the detection of appropriate growing areas (Ma et al. 2022). Using an ANN model, another study explored the synergistic effects of Cd and cerium oxide nanoparticles on rapeseed and their accumulation in diverse plant tissues (Rossi et al. 2019). The ANN model simulated plant uptake of Cd and cerium oxide nanoparticles and recognized significant physiological aspects affecting plant uptake of these elements (Rossi et al. 2019).

Precisely linking genotype information with crop phenotype is a significant challenge in modern breeding, but it is essential for advancing crop improvement programs, especially in the context of metal(loid) tolerance (Harfouche et al. 2019; Esposito et al. 2019; Pazhamala et al. 2021; Khan et al. 2022). Integrating phenomics and HTP with other omics technologies and AI-driven analyses can offer innovative solutions to overcome this challenge and accelerate sustainable agriculture (Fig. 3) (Großkinsky et al. 2018; Raza et al. 2022).

Naika et al. (2013) developed a dataset of stress-responsive signals in *A. thaliana* under various stresses (including Al and Fe toxicity), identifying several shared and unshared biological processes, molecular functions, metabolic pathways, and phenomic traits that may help with the design of stress–smart advanced varieties using genome editing tools. The phenome method was used to detect genetic diversity in root system architecture traits in soybean accessions, revealing similarities in genotype- and phenotype-based clusters, with genotype-based clusters correlated with geographical backgrounds (Falk et al. 2020). While the full potential of phenomics and HTP in breeding crop plants for metal(loid) tolerance has not been explored fully, these techniques offer promising avenues for developing metal(loid)-tolerant crop varieties.

With the help of phenomics and HTP, plant breeders can identify traits that enable crop plants to thrive in metal(loid)-contaminated environments. These traits can be optimized through selective breeding, genome editing, or other advanced techniques. Furthermore, integrating phenomics and HTP with other omics technologies can improve our understanding of how plants respond to metal(loid) toxicity at the molecular level, leading to the identification of novel mechanisms of metal(loid) tolerance and genetic markers that can be exploited in future breeding programs.

## Overview of bio-informatics resources for omics studies

In omics studies, biological databases are uploaded daily with hypothetical/predicted candidate genes, proteins, and metabolites involved in metal(loid) responses. A database survey revealed that some are tailored to specific species or model plants, while others are designed for single stress factors, with only a few versatile enough to encompass various plant species and multiple stress types. Indeed, most omics databases overlook aspects like crop scheduling (systematic planning and management of planting and harvesting times), selection pane features (which could be helpful for agricultural or data visualization purposes), and soil, air, and water-based metal(loid) correlations between laboratory and greenhouse studies. Notably, the output generated from sophisticated instruments and sequencers is multifaceted, diverse, and challenging to consolidate into single or multi-omics-specific databases. The complexity poses a significant technical challenge for computational biologists and data scientists (Misra et al. 2019; Chao et al. 2023), hampering users’ ability to access potential candidates for genetic engineering and genome editing applications.

**Fig. 4:**
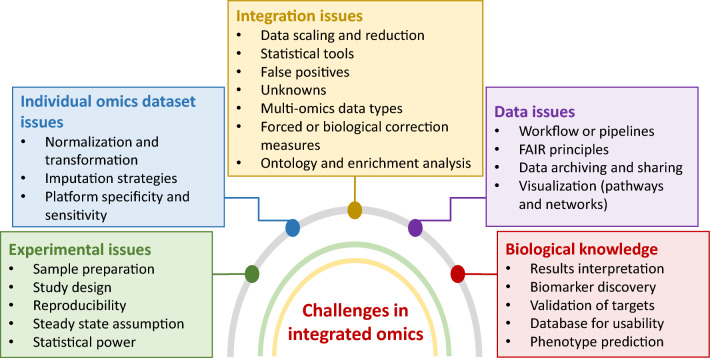
Overview of five major challenges in integrating omics datasets. Modified from Misra et al. (2019)

Given the vast diversity and volume of multi-omics datasets, there is a pressing need for various tools to facilitate data assessment. Numerous omics programs have been developed to address these requirements and streamline the integration of multi-omics datasets (Hernández-de-Diego et al. 2018; Subramanian et al. 2020; Chao et al. 2023). These tools are invaluable for deciphering plant responses and performance across different molecular data stages. Table 4 lists some widely used omics databases and software tools, including their specific functions. Despite the substantial benefits these tools and databases offer, the fast-paced evolution of multi-omics programs has given rise to a lack of standardization among the available resources. Consequently, despite the significant progress in omics analysis, scientists continue to grapple with several major challenges when integrating and analyzing omics datasets (Fig. 4) (Misra et al. 2019; Chao et al. 2023), highlighting the need for dedicated efforts to evaluate and authenticate the characteristics of these resources by scientific communities worldwide.

**Table 4:** List of tools and databases for integrating multi-omics datasets in plants

Tools and databases	Omics integration	Functionality	Future interventions/developments	URL	Reference
Integrated databases					
KaPPa-View4	Metabolomics and transcriptomics	Represents transcriptomics data on metabolic pathway maps	Automated update of data on KaPPA-View4 KEGG	http://kpv.kazusa.or.jp/kpv4/	Sakurai et al. (2011)
MADMAX	Genomics, metabolomics, and transcriptomics	Stores and analyzes multi-omics datasets	Gene expression profiling and improved functional annotation of genes	http://madmax2.bioinformatics.nl/	Lin et al. (2011)
Mix Omics	Metabolomics, metagenomics, proteomics, and transcriptomics	Data exploration and visualization Dimensionality reduction	NP-integration to integrate large-scale data	http://mixomics.org/	Rohart et al. (2017)
MetaboAnalyst 4.0	Metabolomics, metagenomics, and transcriptomics	Data processing and visualization Statistical analysis and functional interpretation	Integration of metabolic sets and pathway libraries of model organisms other than humans Pathway analysis module to be updated to support interactive visual analysis	https://www.metaboanalyst.ca/	Chong et al. (2018)
Plant Metabolic Network	Genomics, metabolomics, and proteomics	Plant-specific databases containing pathways, enzymes, reactions, and compounds	Improved enzyme functional annotation Integrated physical chromosomal span, evolutionary patterns, protein–protein interactions, epigenetic modification marks, and biochemical reactions to better predict plant metabolic gene clusters	https://plantcyc.org/	Pinu et al. (2019)
PlantExp	Transcriptomics and epigenomics	Annotation improvement, multi-way retrieval, and expression analysis	Implementation of differential, specific, coexpression, and cross-species expression analysis to find genes and alternative splicing events	https://biotec.njau.edu.cn/plantExp/	Liu et al. (2023b)
Tools/Software					
Name	Functionality			URL	References
MONGKIE	Enables network analysis and visual mining of multi-omics data			http://yjjang.github.io/mongkie	Jang et al. (2016)
Pathview	Carries out pathway-based data integration and visualization			https://pathview.uncc.edu	Luo et al. (2017)
SLIDE	Performs feature-level and group-level data visualization and allows independent analysis by creating customized gene lists			https://github.com/soumitag/SLIDE	Ghosh et al. (2019)
MapMan	Data visualization and comparative gene expression			https://mapman.gabipd.org/	Schwacke et al. (2019)
MetaBridge	Maps metabolite data to perform pathway visualization and functional analysis with other omics data			https://metabridge.org	Blimkie et al. (2020)
IMPaLA	Integrates pathway knowledge from databases and performs enrichment analysis with metabolite data			http://impala.molgen.mpg.de	Canzler et al. (2020)
Machado	Provides framework to store, browse, and visualize biological data			https://github.com/lmb-embrapa/machado	Mudadu and Zerlotini (2020)
Databases					
MetaCrop	Provides information about metabolic pathways in diverse crop plants and permits automatic exportation of data for the formation of metabolic patterns			https://metacrop.ipk-gatersleben.de/apex/f?p=269:111	Schreiber et al. (2012)
MOPED	Provides information on protein absolute and relative expression data along with gene relative expression data			http://moped.proteinspire.org	Montague et al. (2014)
PlantGenIE	Allows visualization and analysis of genomics and transcriptomics data for diverse plant species			https://plantgenie.org/#	Sundell et al. (2015)
MODEM	Allows genetic mapping and multi-dimensional omics data integration and visualization			http://modem.hzau.edu.cn	Liu et al. (2016)
MetaCyc/BioCyc	Provides information on metabolic pathways and enzymes			https://metacyc.org/ https://biocyc.org/	Caspi et al. (2016)
PlantPReS	Provides information on plant responses to stress conditions at proteome level			http://www.proteome.ir/	Mousavi et al. (2016)
HMOD	Provides a comprehensive set of omics data and KEGG pathway information for herbal medicinal plants			http://herbalplant.ynau.edu.cn	Wang et al. (2018)
KEGG	Provides information on chemicals, genomes, and systemic functional biological pathways			https://www.kegg.jp	Kanehisa et al. (2019)
MaGenDB	Integrates functional annotations at gene, transcript, and protein levels			http://magen.whu.edu.cn	Yang et al. (2020)
Coriander Genomics Database	Allows systematic comparative and evolutionary analyses through cross-species collinearity			http://cgdb.bio2db.com	Song et al. (2020)
ZEAMAP	Provides information on genes and comparative expression patterns			http://www.zeamap.com	Gui et al. (2020)
GERDH	Provides information on gene expression and biological functions across horticultural plants			https://dphdatabase.com	Cheng et al. (2023a)
SCIPDb	Provides comprehensive information for understanding combined stress responses in different plants			http://www.nipgr.ac.in/scipdb.php	Priya et al. (2023)

## Concluding remarks and future outlook

In this comprehensive review, we have meticulously examined the current literature, focusing on three major omics techniques—transcriptomics, proteomics, and metabolomics—pivotal for advancing crop improvement programs to develop metal(loid)-tolerant plants. Beyond delving into the insights these techniques offer individually, we have explored the potential of integrating them to gain a deeper understanding of common stress-tolerant mechanisms. Throughout this review, we have highlighted recent advances, such as stress-tolerant candidate DEGs, key regulatory signaling, molecular, and metabolic pathways, and the myriad of specific and non-specific genes, proteins, metabolites, and intermediate products responsive to metal(loid) stress, illustrating these insights with the latest examples from various plant types. Furthermore, we have emphasized the importance of bridging classical breeding approaches with omics techniques to grasp the mechanistic underpinnings of prevailing changes or regulations during metal(loid) exposure and plant–environment interactions. This dual strategy enables us to unravel the intricate network of metabolic pathways and metabolites that govern genotype-to-phenotype changes in metal(loid)-stressed plants. Omics studies are vital in narrowing the gap between laboratory research and field applications, facilitating the development of stress-tolerant crop varieties with desirable agronomic traits and high yields. Moreover, we highlighted advances in high-tech instruments and computational biology tools that facilitate big data collection for single or multi-omics approaches. One noteworthy observation is the continuous influx of data into databases, with a substantial volume of predictable potential stress-responsive genes at various stages of plant development under metal(loid) stress. However, the challenge for data scientists remains in handling, maintaining, retrieving, mapping, and presenting the connectivity and inter-relationships among genes, proteins, and metabolites. With our increased understanding of information technology like ML, AI, and data analysis pipelines, the impact of omics platforms on crop improvement and breeding programs becomes increasingly profound in environmental stress biology research.

Integrating omics data with AI and HTP or phenomics holds immense potential for revolutionizing crop breeding and yields (Fig. 3). ML algorithms now enable precise associations between genotypes and phenotypes, expediting the discovery of genes linked to specific traits such as metal(loid) tolerance. This approach can help fast-track breeding programs, resulting in the development of stress–smart crop varieties that can withstand multiple stresses, including metal(loid) toxicity. Future directions should focus on advancing more sophisticated AI algorithms and seamlessly integrating multi-omics data to better understand plant stress biology and identify more efficient targets for crop improvement. Importantly, efforts should be made to ensure these emerging tools are accessible and affordable to small-scale farmers, especially in developing countries where crop improvement is crucial to meet food demands.

As a computational approach, omics presents technical challenges that must be addressed. Currently, databases tend to be model organism-specific, limiting the correlation between experimental and analytical studies across plant species that are constantly evolving. Moreover, in nature, plants often face multiple stress factors alongside co-contaminants. Multi-omics databases need to capture the intensity and versatility of such stress conditions. Each database has specific data filtering, interpretation, and mapping interfaces, making it challenging to represent data uniformly in single or multi-omics databases. As a result, mathematical modeling and prediction from laboratory studies demand skilled and trained data scientists, a resource that is lacking globally. This lack of resources can be attributed to factors such as limited awareness, inadequate infrastructure, insufficient capital investments, and reduced funding support from governing bodies. The field of systems biology is advancing omics approaches by integrating information from various omics databases for single, coupled, or multiple stress factors, bringing them onto a common platform to understand plant stress relationships holistically in contaminated environments. Significant refinements in bioinformatics databases, tools, and pipelines, alongside traditional breeding and field studies, are essential and critically needed for identifying potential stress-responsive candidates. These identified stress-related players can then be genetically engineered to develop sustainable, stress–smart, and nutritionally rich crops for future generations.

## Funding

This work was supported by startup grants from the Food Futures Institute of Murdoch University, Australia, to RKV.

## Declarations

### Data availability

This is a review paper; all resources used are cited correctly.
